# Profiling of RNA *N*^*6*^-Methyladenosine Methylation Reveals the Critical Role of m^6^A in Chicken Adipose Deposition

**DOI:** 10.3389/fcell.2021.590468

**Published:** 2021-02-05

**Authors:** Bohan Cheng, Li Leng, Ziwei Li, Weijia Wang, Yang Jing, Yudong Li, Ning Wang, Hui Li, Shouzhi Wang

**Affiliations:** ^1^Key Laboratory of Chicken Genetics and Breeding, Ministry of Agriculture and Rural Affairs, Harbin, China; ^2^Key Laboratory of Animal Genetics, Breeding and Reproduction, Education Department of Heilongjiang Province, Harbin, China; ^3^College of Animal Science and Technology, Northeast Agricultural University, Harbin, China

**Keywords:** chicken, fat deposition, adipose tissue, *N*^6^-methyladenosine, MeRIP-seq

## Abstract

One of the main objectives of broiler breeding is to prevent excessive abdominal adipose deposition. The role of RNA modification in adipose deposition is not clear. This study was aimed to map m^6^A modification landscape in chicken adipose tissue. MeRIP-seq was performed to compare the differences in m^6^A methylation pattern between fat and lean broilers. We found that start codons, stop codons, coding regions, and 3′-untranslated regions were generally enriched for m^6^A peaks. The high m^6^A methylated genes (fat birds vs. lean birds) were primarily associated with fatty acid biosynthesis and fatty acid metabolism, while the low m^6^A methylated genes were mainly involved in processes associated with development. Furthermore, we found that the mRNA levels of many genes may be regulated by m^6^A modification. This is the first comprehensive characterization of m^6^A patterns in the chicken adipose transcriptome, and provides a basis for studying the role of m^6^A modification in fat deposition.

## Introduction

As a result of long-term breeding efforts, the growth rate and meat yield of broilers have significantly improved; however, this has led to excessive body fat (especially abdominal fat) deposition. The accumulation of excess fat in broilers has many undesirable consequences, such as decreased reproductive performance and reduced feed-conversion efficiencies (Zhou et al., 2006; Zhang et al., 2018). Adipose tissue is an important energy storage and endocrine organ and is the cornerstone of energy metabolism homeostasis (McGown et al., 2014; Choe et al., 2016). Adipose tissue development is controlled by a complex network of transcription factors (Farmer, 2006). In addition to transcriptional regulation, evidence suggests that adipose development and fat deposition can be regulated by the epigenetic mechanisms, such as DNA methylation (Zhu et al., 2012), histone modification (Wang et al., 2010), and chromatin remodeling (Siersbaek et al., 2011).

In addition to the chemical modification of DNA and proteins, RNA modification has become a research hotspot in the field of epigenetics in recent years. So far, more than 100 types of chemical modifications of RNA have been identified, with *N*^6^-methyladenosine (m^6^A) methylation being the most pervasive modification in eukaryotes (Yue et al., 2015). M^6^A is installed by a multicomponent methyltransferase complex consisting of Methyltransferase Like 3 (METTL3), METTL14 and Wilms Tumor 1 Associated Protein (WTAP), and erased by m^6^A demethylase fat mass and obesityassociated protein (FTO) and α-ketoglutarate-dependent dioxygenase alkB homolog 5 (ALKBH5) (Yang et al., 2018). M^6^A is involved in many important biological processes through the post-transcriptional regulation of gene expression, including mRNA export, the processing of pri-miRNA, alternative splicing, mRNA degradation and translation (Yang et al., 2018).

In mammals, emerging evidence shows that m^6^A modification plays a critical role in adipose development and hepatic lipid metabolism (Tao et al., 2017; Lu et al., 2018; Wang et al., 2018). Knockdown of METTL3, METTL14, WTAP or FTO inhibited the differentiation of mouse 3T3-L1 preadipocytes (Zhao et al., 2014; Kobayashi et al., 2018). However, whether m^6^A modification is involved in poultry adipose deposition is still largely unknown. Here, we used Northeast Agricultural University broiler lines divergently selected for abdominal fat content (NEAUHLF) as fat and lean animal models to compare the differences in m^6^A topological patterns and functions. We collected abdominal adipose tissue from the two broiler lines for m^6^A methylation profiling with methylated RNA immunoprecipitation (IP) sequencing (MeRIP-seq). Our data showed that the adipose tissue mRNA was extensively methylated with m^6^A to fine-tuning the expression of genes responsible for lipid metabolism and adipogenesis.

## Materials and Methods

### Experimental Birds and Management

Animal studies were conducted according to the guidelines for the care and use of experimental animals established by the Ministry of Science and Technology of the People's Republic of China (approval number: 2006-398) and were approved by the Laboratory Animal Management Committee and the Institutional Biosafety Committee of Northeast Agricultural University (Harbin, China). In total, six male birds (lean line, *n* = 3, and fat line, *n* = 3) from the 23rd generation (G_23_) of NEAUHLF were used for MeRIP-seq analysis. NEAUHLF has been selected since 1996 using plasma very-low-density lipoprotein concentration and abdominal fat percentage (AFP; abdominal fat weight [AFW]/body weight at 7 weeks [wk] of age [BW_7_]) as selection criteria. Details of the breeding procedure have been described previously (Guo et al., 2011; Zhang et al., 2017). All birds used in this study were kept in similar environmental conditions and had free access to feed and water. From hatching to 3 wk of age, all birds received the starter feed (3,100 kcal of ME/kg and 210 g/kg of crude protein [CP]).Then, from 4 to 7 wk of age, all birds were fed a grower diet (3,000 kcal of ME/kg and 190 g/kg of CP).

### Tissue Collection

Six male birds (three birds of each broiler line at 7 wk of age) from G_23_ were slaughtered after fasting for 10 h, and the BW_7_ and AFW were measured and used to calculate AFP (Supplementary Figure 1). Abdominal fat tissues were collected, washed with 0.75% NaCl solution, snap-frozen in liquid nitrogen, and stored at −80°C until RNA extraction.

### RNA Isolation and Fragmentation

The total RNA from the abdominal adipose tissue was extracted using TRIzol reagent (Invitrogen Co., CA, USA) according to the manufacturer's instructions. The ribosomal-RNA content of the total RNA was reduced using the Ribo-Zero rRNA Removal Kit (Illumina Inc., CA, USA). Then, the RNA was chemically fragmented into fragments of ~100 nucleotides in length using fragmentation buffer (Illumina Inc.).

### Methylated RNA IP Library Construction and Sequencing

The MeRIP-seq service was provided by Cloudseq Biotech Inc. (Shanghai, China). Briefly, IP of the m^6^A RNA was performed with the GenSeq^TM^ m^6^A RNA IP Kit (GenSeq Inc., China) following the manufacturer's instructions. Both the m^6^A IP samples and the input samples without IP were used for library generation with NEBNext® Ultra II Directional RNA Library Prep Kit (New England Biolabs Inc., USA). The quality of the libraries was evaluated with the BioAnalyzer 2100 system (Agilent Technologies Inc., USA). Library sequencing was performed on an Illumina Hiseq instrument with 150 bp paired-end reads.

### MeRIP-seq Analysis

Briefly, paired-end reads were harvested from the Illumina HiSeq 4000 sequencer, and Q30 scoring was used for quality control. Cutadapt software (v1.9.3) was employed for 3′-adaptor-trimming and removal of low-quality reads. The clean reads were aligned to the reference chicken genome sequences (galGal6) using Hisat2 software (v2.0.4), and only the uniquely mapped and non-duplicated alignments were further analyzed. The m^6^A-modification peaks were called by MACS software (Zhang et al., 2008), the “effective genome size” parameter was adjusted to the calculated transcriptome size (1.77 × 10^8^) (Dominissini et al., 2012); meanwhile, the input RNA sequencing (RNA-seq) data were used as the background when calling peaks. Peaks that shared at least 50% overlapping lengths were defined as recurrent peaks.

To examine the distribution pattern of the m^6^A peaks throughout different regions of the transcripts, the mRNA transcripts were divided into five non-overlapping segments: the 5′UTR, start codon (100 nucleotides centered on the start codon), CDS, stop codon (100 nucleotides centered on the stop codon), and 3′UTR. Each area was separated into 20 bins (Luo et al., 2014). Bedtools (v2.26.0) was used to count the peak number of each bin, and the counts were employed to plot the patterns by R (v3.4.4).

In the fat and lean groups, the top 1,000 significantly enriched peaks (MACS-assigned fold change > 2 and *P* < 0.00001) within the mRNAs from three biological replicates were combined (Dominissini et al., 2012), and 101 nucleotides centered on the collected peaks of each group were subjected to *de novo* motif analysis using DREME software (Bailey, 2011). The DREME tool in the MEME suite (http://meme-suite.org/tools/dreme) was used to discover relatively short (up to 8 bp), ungapped motifs that are enriched within a set of target sequences (m^6^A peak sequences) relative to a set of control sequences (shuffled m^6^A peak sequences) (Bailey, 2011).

We obtained the common and unique peaks using bedtools intersect (v2.26.0). For a peak to be classified as line-unique, it was assumed not to overlap (<50% overlapping length) any peak of the other line; meanwhile, we defined a peak that appeared in both chicken lines as a common peak (≥50% overlapping length). Line-dynamic methylated peaks (fold change ≥2 and *P* < 0.05), which showing a change of intensity in some of the common peaks, were identified by diffReps software.

The gene expression levels were determined using the input data, and the number of sequenced fragments of each transcript was normalized using the algorithm of Fragments Per Kilobase of Transcript Per Million Fragments Mapped (FPKM) by Cufflinks software (v2.2.1). Differentially expressed transcripts (fold change ≥2 and *P* < 0.05) between fat and lean groups were identified with the Cuffdiff program (v2.2.1). The FPKM of input and IP samples were calculated by Cufflinks software (v2.2.1). NNFPKM (NNFPKM = FPKM_IP/FPKM_INPUT) were used to analyze the m^6^A enrichment level in fat and lean groups. Then FPKM_INPUT and NNFPKM were log2 transformed and Pearson correlation analysis of mRNA expression levels and m^6^A methylation levels was carried out. Gene Ontology (GO) and pathway analyses were performed using GO (www.geneontology.org) and the Kyoto Encyclopedia of Genes and Genomes (KEGG) database (www.genome.jp/kegg). The thresholds for significant enrichment for GO and KEGG analysis were set at *P* < 0.05.

## Results

### Transcriptome-Wide Mapping of the M^6^A Methylation Landscape in Chicken Adipose Tissues

MeRIP-seq produced 80,966,354–102,649,284 raw reads from input or IP abdominal fat tissues from lean line (L-AF) and fat line (F-AF) chickens. After filtering out low quality data, more than 71,600,000 high-quality reads from each sample were mapped to the galGal6 genome. More than 86% of the clean reads from all the samples were uniquely mapped to chicken reference genome (Table 1). In the input samples, we detected 9,041 and 9,452 expressed mRNA transcripts in lean line and fat line, respectively. After the methylated-RNA fragments were mapped to the transcriptome, 4,615 m^6^A transcripts (common m^6^A transcripts from three biologic replicates) were identified among the 5,965 coding transcripts (common mRNA transcripts from three biologic replicates) in the L-AF samples and 4,438 m^6^A transcripts among the 6,654 coding transcripts in the F-AF samples (Table 2). The proportion of methylated transcripts were 77 and 67% in L-AF and F-AF, respectively. In addition, we detected 7,097 recurrent m^6^A peaks within 5,965 coding transcripts in L-AF and 6,966 recurrent m^6^A peaks among 6,654 coding transcripts in F-AF (Table 2; Supplementary Data 1). Based on this information, we estimated that the chicken abdominal adipose transcriptome contained 1.54–1.57 m^6^A peaks per methylated transcript and 1.05–1.19 m^6^A peaks per transcript. These data are similar to those of the chicken ovary and pig *longissimus dorsi* muscle, which have ~1.5 m^6^A peaks per methylated transcript (Fan et al., 2019; Jiang et al., 2019); and mouse liver and pig subcutaneous fatty tissue transcriptomes, which possess 1.3 m^6^A peaks per transcript (Dominissini et al., 2012; Tao et al., 2017). However, our results were lower than that of the mouse L cells, which presenting about 3 m^6^A residues per mRNA transcript (Perry et al., 1975).

**Table 1:** Summary of sequencing data and read-alignment statistics from MeRIP-seq in abdominal adipose in fat and lean broiler lines.

**Sample ID**	**Raw reads**	**Clean reads**	**Reads uniquely mapped to genome**	**Clean reads uniquely mapped (%)**
L-AF-IP	93,333,165	93,228,190	80,368,297	86.21
L-AF-input	91,232,892	90,901,841	80,641,851	88.71
F-AF-IP	102,649,284	102,539,687	89,675,215	87.46
F-AF-input	80,966,354	80,583,035	71,641,685	88.81

**Table 2:** Number of m^6^A peaks detected in the abdominal adipose of the two chicken lines.

**Sample ID^a^**	**mRNA transcripts**	**m^**6**^A mRNA transcripts**	**Total m^**6**^A peaks**	**Total m^**6**^A peaks per m^**6**^A transcript**	**Total m^**6**^A peaks per transcript**
L-AF-1	7,639	6,563	12,164	1.85	1.59
L-AF-2	7,965	6,408	11,841	1.85	1.49
L-AF-3	6,914	6,642	12,919	1.95	1.87
L-AF	5,965	4,615	7,097	1.54	1.19
F-AF-1	8,634	6,170	12,015	1.95	1.39
F-AF-2	7,463	6,444	12,958	2.01	1.74
F-AF-3	8,023	6,017	11,307	1.88	1.41
F-AF	6,654	4,438	6,966	1.57	1.05

a *L-AF-1,L-AF-2, and L-AF-3 refer to sample 1, sample 2, and sample 3 of abdominal adipose tissue from lean line, respectively; L-AF means the recurrent peak sample for L-AF-1, L-AF-2, and L-AF-3 (≥50% overlapping lengths); F-AF-1,F-AF-2, and F-AF-3 refer to sample 1, sample 2, and sample 3 of abdominal adipose tissue from fat line, respectively; F-AF means the recurrent peak sample for F-AF-1, F-AF-2, and F-AF-3 (≥50% overlapping lengths)*.

**Figure 1:**
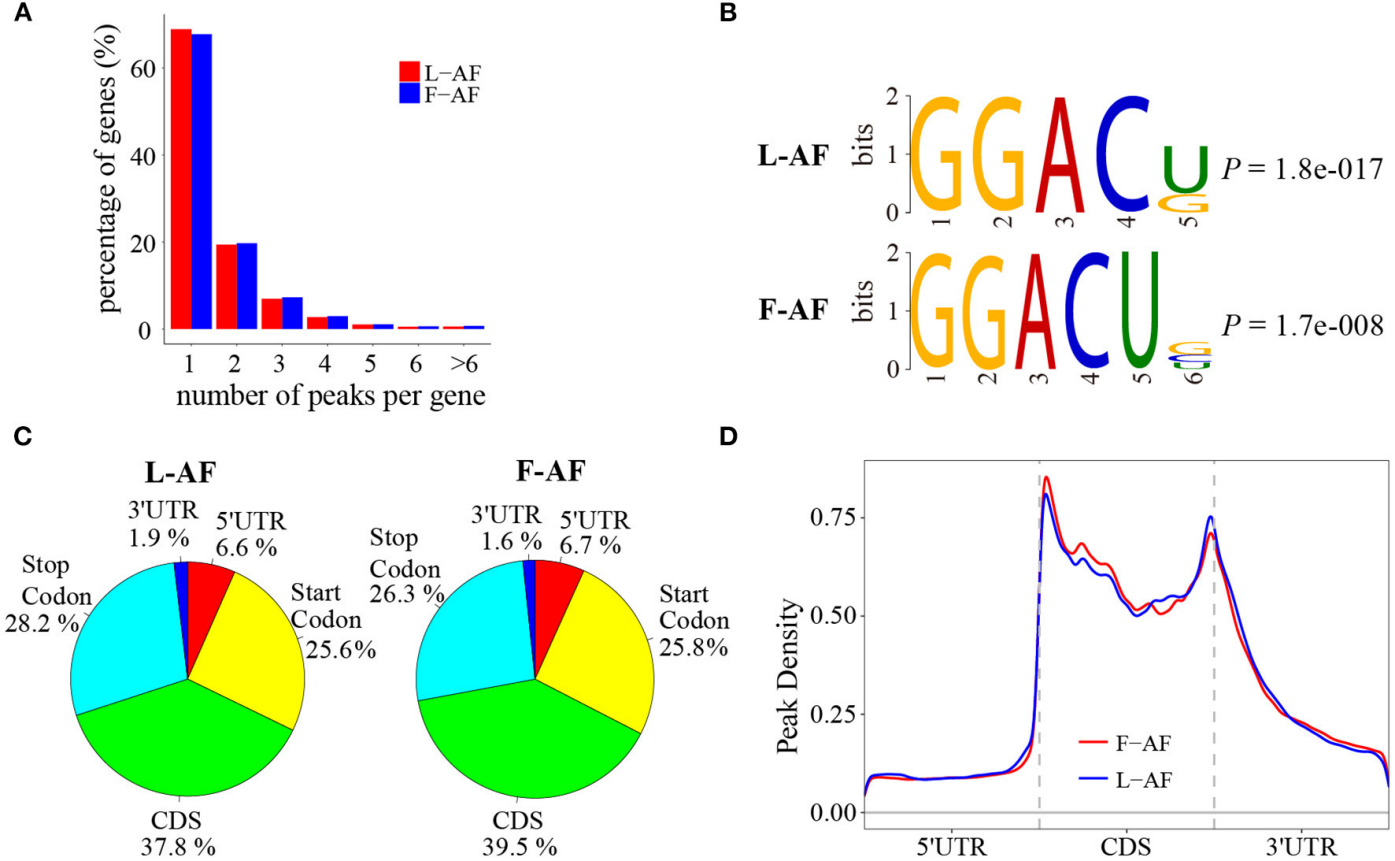
Overview of *N*^6^-methyladenosine methylation within mRNAs in fat- and lean-line broiler chickens. **(A)** Sequence motif of m^6^A-containing peak regions. **(B)** Percentage of m^6^A-methylated transcripts with different number of m^6^A peaks. **(C)** Pie charts showing the percentage of m^6^A peaks in five non-overlapping segments of mRNA transcripts. **(D)** Enrichment of m^6^A peaks along mRNA transcripts. L-AF and F-AF represent abdominal fat tissue from lean- and fat-line chickens, respectively.

To determine how the m^6^A modification was distributed throughout the chicken transcriptome. We classified the methylated transcripts based on the number of m^6^A peaks contained in each transcript, and found that nearly 85% of the methylated transcripts contained one or two m^6^A peaks, and about 5% of the methylated transcripts contained four or more peaks (Figure 1A); this ratio is similar to that previously reported in humans (5.5%) (Dominissini et al., 2012) but is lower than that in pigs (10%) (Wang et al., 2018) and *Arabidopsis thaliana* (17%) (Wan et al., 2015). We then investigated whether the m^6^A peaks we identified share a conservative RRACH motif (where R stands for purine, A represents m^6^A, and H represents a non-guanine base) (Dominissini et al., 2012; Meyer et al., 2012; Luo et al., 2014), we conducted a search for the motifs enriched in the regions around the m^6^A peaks. The results showed that GGACU was significantly enriched and consistently considered to be the best motif in both broiler lines (Figure 1B). To confirm the preferential localization of m^6^A in the transcripts, m^6^A peaks were categorized into five non-overlapping segments: 5′UTR, the start codon segment, coding sequence (CDS), the stop codon segment and 3′UTR. Our results show that m^6^A was most often located in the CDS, and sometimes near the start and stop codons (Figure 1C), which is consistent with the patterns identified in the mouse and pig (Tao et al., 2017; Luo et al., 2019). Metagene profiling of the m^6^A peaks showed that they were primarily enriched in CDSs, near the start and stop codons, and close to the beginning of 3′UTRs (Figure 1D), which differs from the pattern identified in mammals (Meyer et al., 2012; Tao et al., 2017).

### Biological Pathways Associated With Common and Line-Unique M^6^A Genes

**Figure 2:**
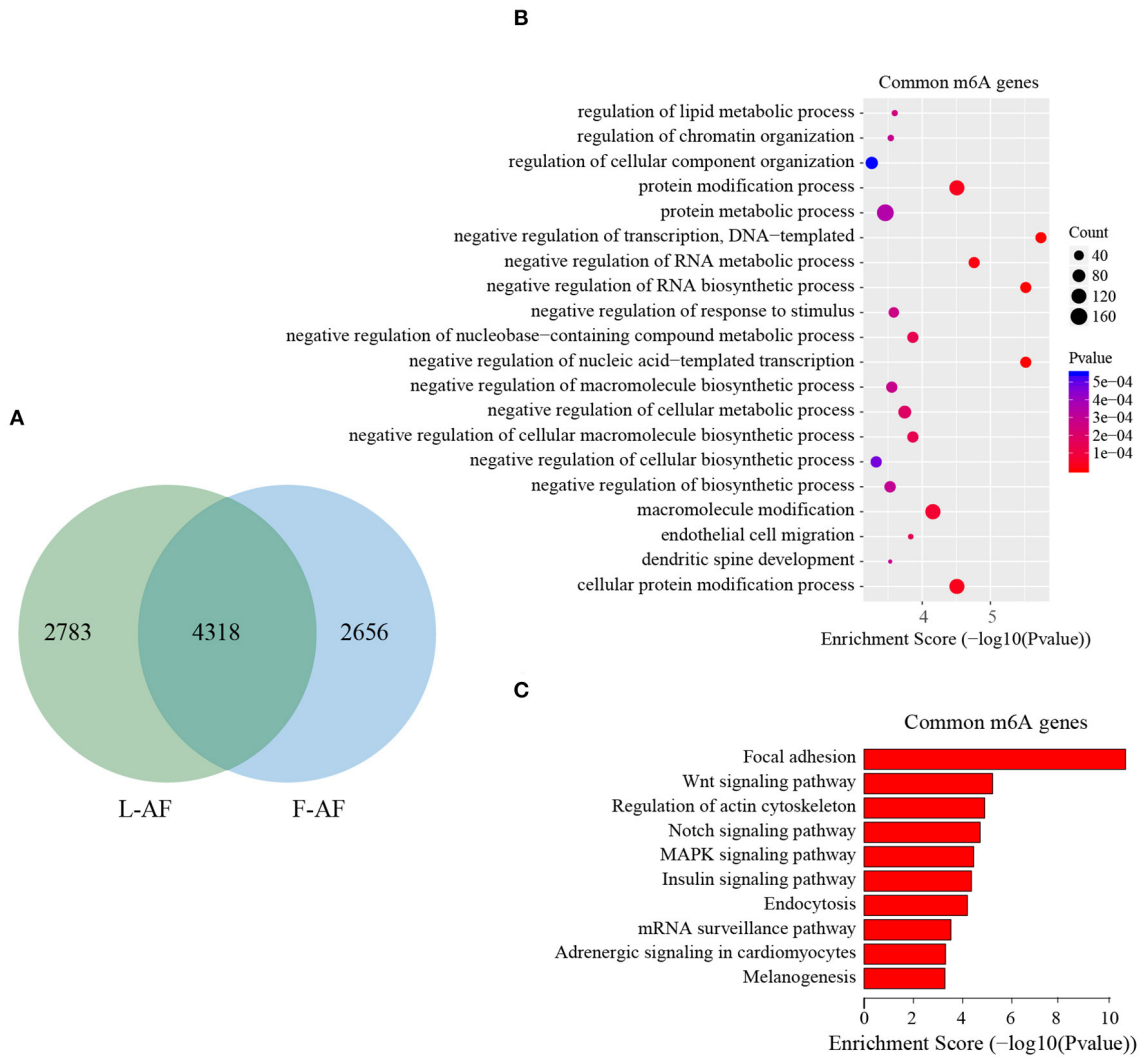
GO biological process and KEGG pathway analyses of common m^6^A genes in broiler chickens. **(A)** Venn diagram showing overlap of the m^6^A peaks from L-AF and F-AF samples. **(B)** GO enrichment analysis of common m^6^A genes (*P* < 0.05). **(C)** Pathway analysis of common m^6^A genes (*P* < 0.05).

**Figure 3:**
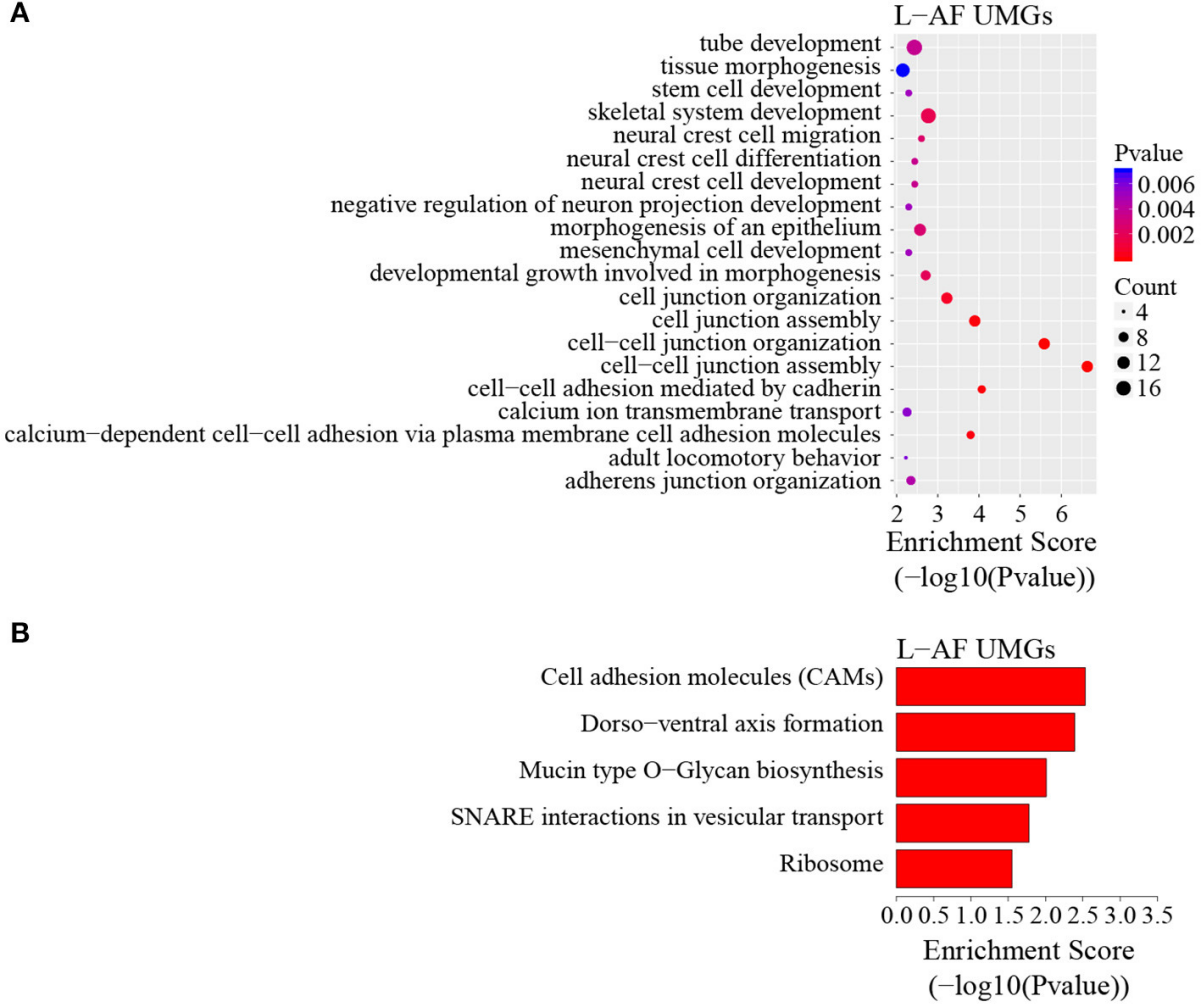
GO biological process and KEGG pathway analyses of unique m^6^A genes in lean-line broiler chickens. **(A)** GO analysis of unique m^6^A genes in the lean line (*P* < 0.05). **(B)** Pathway analysis of unique m^6^A genes in the lean line (*P* < 0.05).

**Figure 4:**
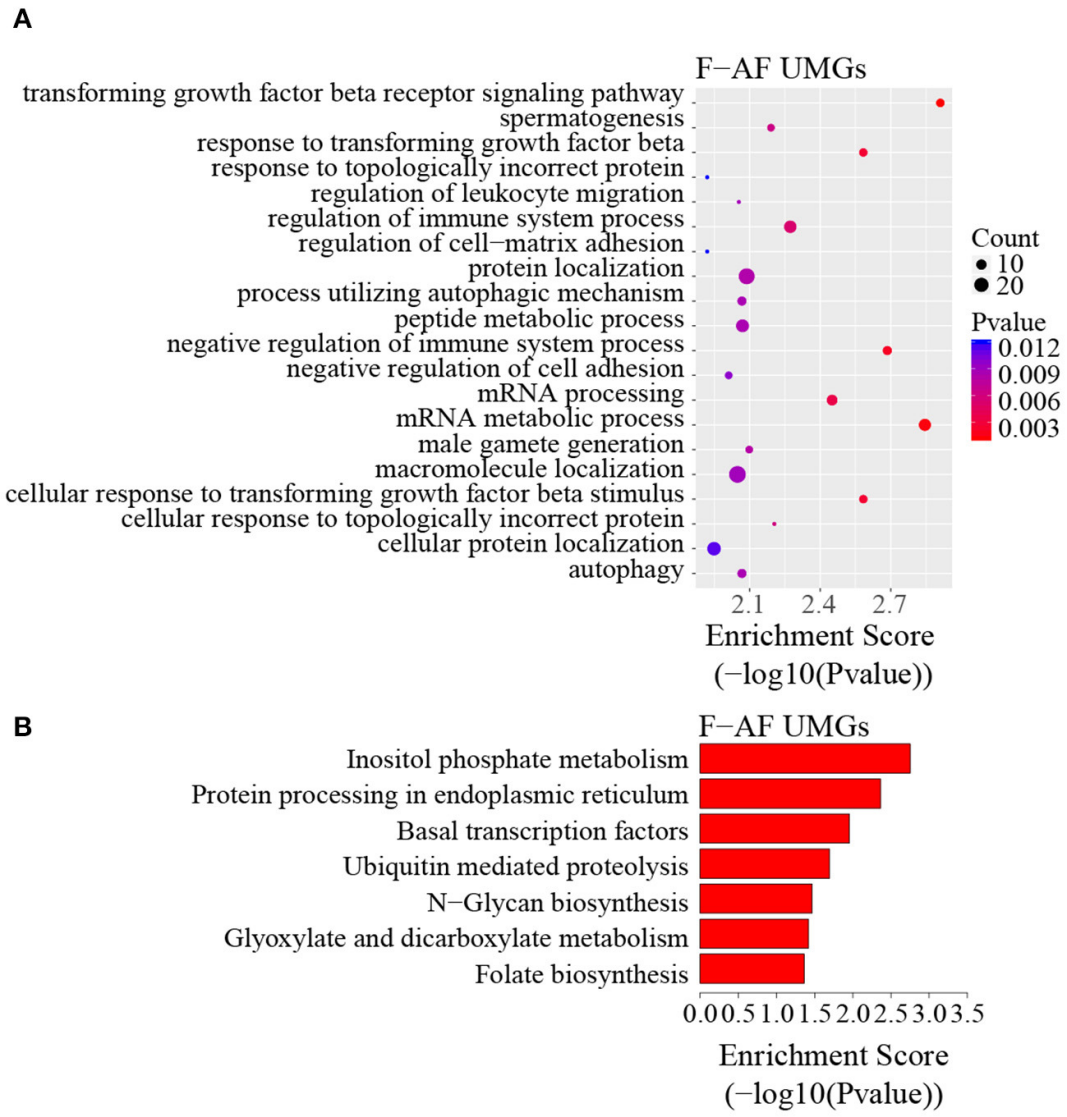
GO biological process and KEGG pathway analyses of unique m^6^A genes in fat-line broiler chickens. **(A)** GO analysis of unique m^6^A genes in the fat line (*P* < 0.05). **(B)** Pathway analysis of unique m^6^A genes in the fat line (*P* < 0.05).

To discover the differencesin m^6^A modification between the two chicken lines, we first identified the line-unique m^6^A peaks and genes. We found 4,318 peaks (representing 3,325 genes) that were common methylated in L-AF and F-AF, along with 2,783 and 2,656 peaks (representing 1,290 and 1,113 genes, respectively) that were specifically methylated in L-AF and F-AF, respectively (Figure 2A; Supplementary Data 2). To predict the functions associated with the m^6^A-modified genes, we conducted the gene ontology (GO) biological process (BP) and KEGG pathway analysis. The m^6^A genes common to both lines were predominantly assigned to lipid metabolism, transcription, protein modification, the Wnt-signaling pathway, and the cytoskeleton (*P* < 0.05, Figures 2B,C, Supplementary Data 3, 4). In addition, the L-AF-unique m^6^A genes (L-AF UMGs) were significantly involved in development-associated processes, cell junction assembly, ribosome biogenesis, and others (*P* < 0.05, Figures 3A,B, Supplementary Data 3, 4). However, the F-AF-unique m^6^A genes (F-AF UMGs) were generally involved in the cellular responses to transforming growth factor-beta, mRNA processing, protein localization, and ubiquitin-mediated proteolysis (*P* < 0.05, Figures 4A,B, Supplementary Data 3, 4).

### Involvement of Line-Dynamic M^6^A Genes in Lipogenesis-Related Pathways

**Figure 5:**
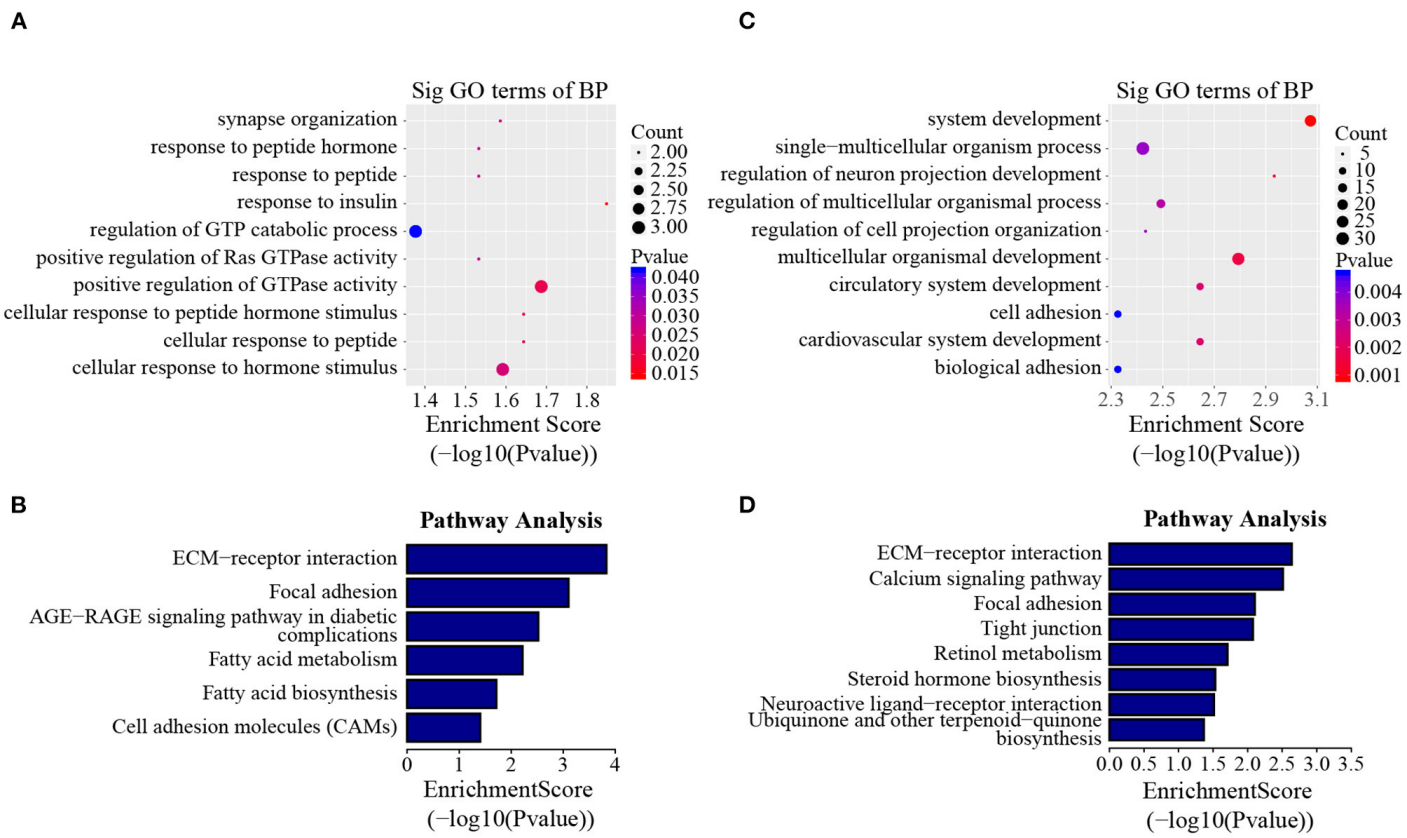
GO biological process and KEGG pathway analyses of line-dynamic m^6^A genes. **(A)** GO analysis of high m^6^A methylated genes (fat line vs. lean line; *P* < 0.05). **(B)** Pathway analysis of high m^6^A methylated genes (fat line vs. lean line; *P* < 0.05). **(C)** GO analysis of low m^6^A methylated genes (fat line vs. lean line; *P* < 0.05). **(D)** Pathway analysis of low m^6^A methylated genes (fat line vs. lean line; *P* < 0.05). BP, biological process; Sig, significant.

In addition to the line-unique m^6^A genes, the line-dynamic m^6^A genes (common to both chicken lines but with different m^6^A peak intensities) were also selected for GO biological process and KEGG pathway analyses. We found 1,504 common m^6^A peaks with remarkably different abundances between the two chicken lines, which represented 1,172 coding genes, of which 71.1% (1,069/1,504) were lower in the fat line compared with the lean line (Table 3, Supplementary Data 5). Tables 4, 5 show the top 15 high and low m^6^A peaks of mRNAs (fat line vs. lean line) with the highest fold-change values. We discovered that the genes with high m^6^A peaks were mainly involved in the cellular responses to peptide hormone stimuli and lipogenesis-related pathways, including fatty acid biosynthesis and fatty acid metabolism (*P* < 0.05, Figures 5A,B, Supplementary Data 6, 7), while those with low m^6^A peaks were involved with development-associated processes, calcium-signaling pathway, steroid hormone biosynthesis, and others (*P* < 0.05, Figures 5C,D, Supplementary Data 6, 7).

**Table 3:** General numbers of line-dynamic methylated peaks and associated genes.

**High methylated peak^a^**	**High methylated gene^a^**	**Low methylated peak^a^**	**Low methylated gene^a^**
435	334	1,069	838

a *Fat line vs. lean line*.

**Table 4:** Top 15 high m^6^A peaks and associated genes.

**Chromosome**	**txStart^a^**	**txEnd^b^**	**Gene name**	**Gene description**	**Fold change^c^**
11	15359661	15360020	CMC2	Cytochrome c oxidase biogenesis	231.6
3	66300750	66301060	RPF2	Ribosomal large subunit assembly	94.2
12	17381938	17382188	CNTN3	Nervous development	87.1
1	51607928	51608248	NCF4	NADPH-oxidase complex assembly	86.4
Z	53573841	53574300	PDE6B	Signal transduction	84.4
8	28154046	28154232	ALG6	Glycosylation of lipids	78.0
5	5892541	5892820	QSER1	Nervous development	73.4
6	11187017	11187280	MYPN	Muscle contraction	57.9
6	18326706	18326912	SCD	Fatty acid biosynthesis	52.3
1	245141	246156	CD69L	Cell adhesion	44.7
23	4068703	4068954	GRIK3	Nervous development	32.7
3	71489501	71489822	PNISR	Pre-mRNA splicing	26.1
3	18088321	18088560	BROX	Protein ubiquitination	22.0
16	2069078	2069235	MICA	Immune response	20.3
7	23307358	23307791	IGFBP2	Signal transduction	20.1

a *Start position of the high m^6^A peaks (fat line vs. lean line)*.

b *End position of the high m^6^A peaks (fat line vs. lean line)*.

c *Ratio of m^6^A peak intensity in the fat line relative to the lean line*.

**Table 5:** Top 15 low m^6^A peaks and associated genes.

**Chromosome**	**txStart^a^**	**txEnd^b^**	**Gene name**	**Gene description**	**Fold change^c^**
5	15161823	15162078	MUC2	O-glycan processing	2471.4
5	15157562	15157817	MUC2	O-glycan processing	1162.5
2	149536521	149536599	LOC107050437	Novel gene	1012.1
2	149532830	149533180	LOC107050437	Novel gene	616.0
20	9004330	9004481	EEF1A2	Elongation of translation	507.8
18	616601	616800	MYH1C	Muscle contraction	453.1
5	51897	52030	LOC107051134	Novel gene	435.8
3	22465749	22466142	KCNH1	Ion transport	431.3
27	7062978	7063300	GRB7	Signal transduction	413.1
8	24407796	24408005	TTC39A	Type 2 diabetes	363.5
3	59176501	59177047	SOGA3	Autophagy	362.8
14	6410912	6411084	MSLN	Cell adhesion	343.6
6	32348438	32348614	LOC112532717	Novel gene	275.2
6	5199540	5199624	SFTPA2	Surfactant-related functions	261.0
Z	10819061	10819246	SPEF2	Sperm axoneme assembly	239.2

a *Start position of the low m^6^A peaks (fat line vs. lean line)*.

b *End position of the low m^6^A peaks (fat line vs. lean line)*.

c *Ratio of m^6^A peak intensity in the lean line relative to the fat line*.

### Gene mRNA-Level Regulation by m^6^A Modification

**Figure 6:**
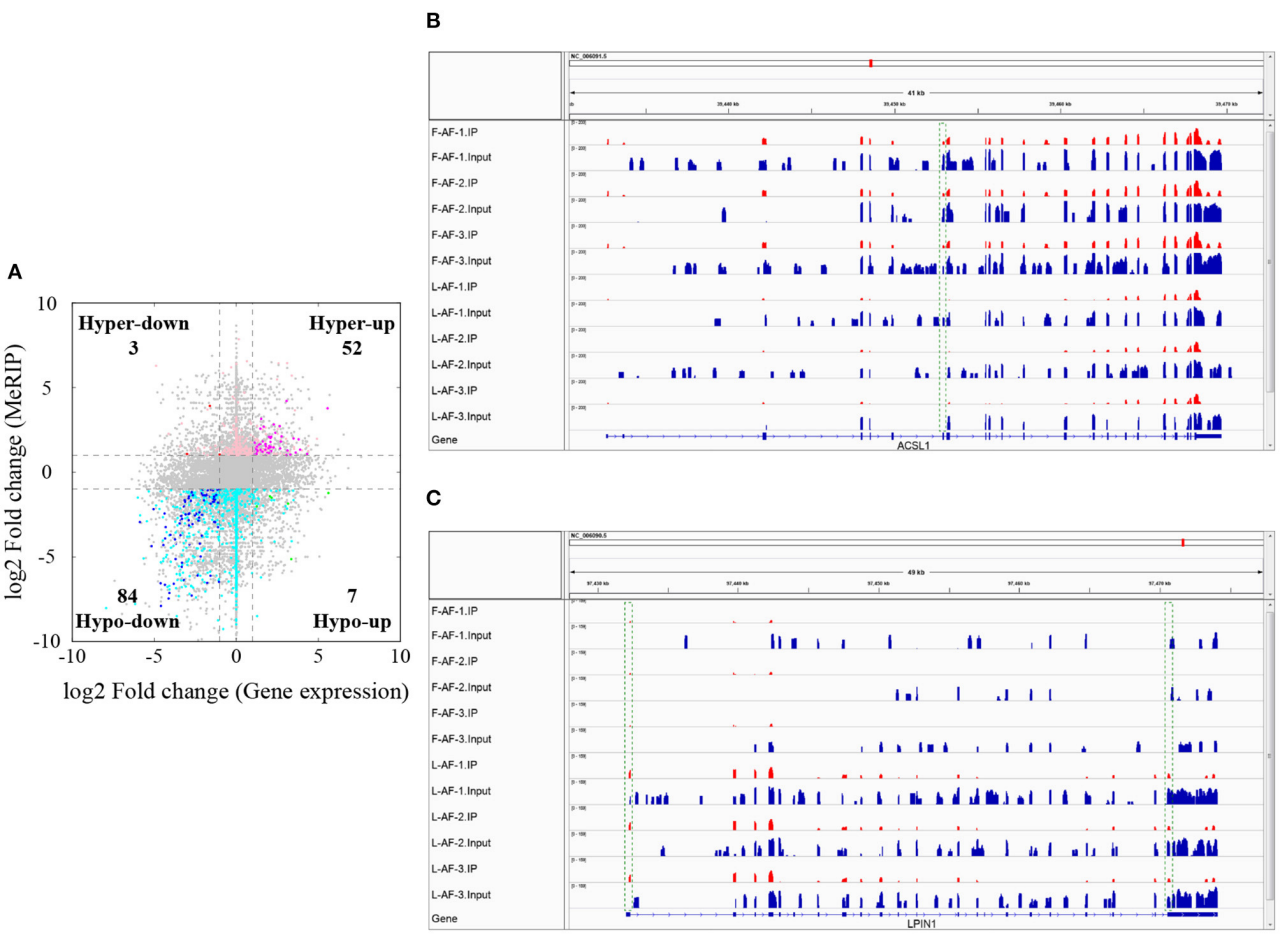
Relationship between m^6^A content and mRNA levels in adipose tissue of broiler chickens. **(A)** Distribution of genes with a marked change in both mRNA expression and m^6^A methylation in fat-line compared with lean-line chickens. **(B)** The abundance of m^6^A in the *ACSL1* mRNA transcripts of fat (F-AF-1, F-AF-2, and F-AF-3) and lean (L-AF-1, L-AF-2, and L-AF-3) birds, as detected by MeRIP-seq. The intensity of the m^6^A peak shown in the green rectangle is markedly higher in fat birds compared with lean birds. **(C)** The abundance of m^6^A in the *LPIN1* mRNA transcript in fat and lean birds, as detected by MeRIP-seq. The intensities of the m^6^A peaks shown in the green rectangles are markedly lower in fat birds compared with lean birds.

**Figure 7:**
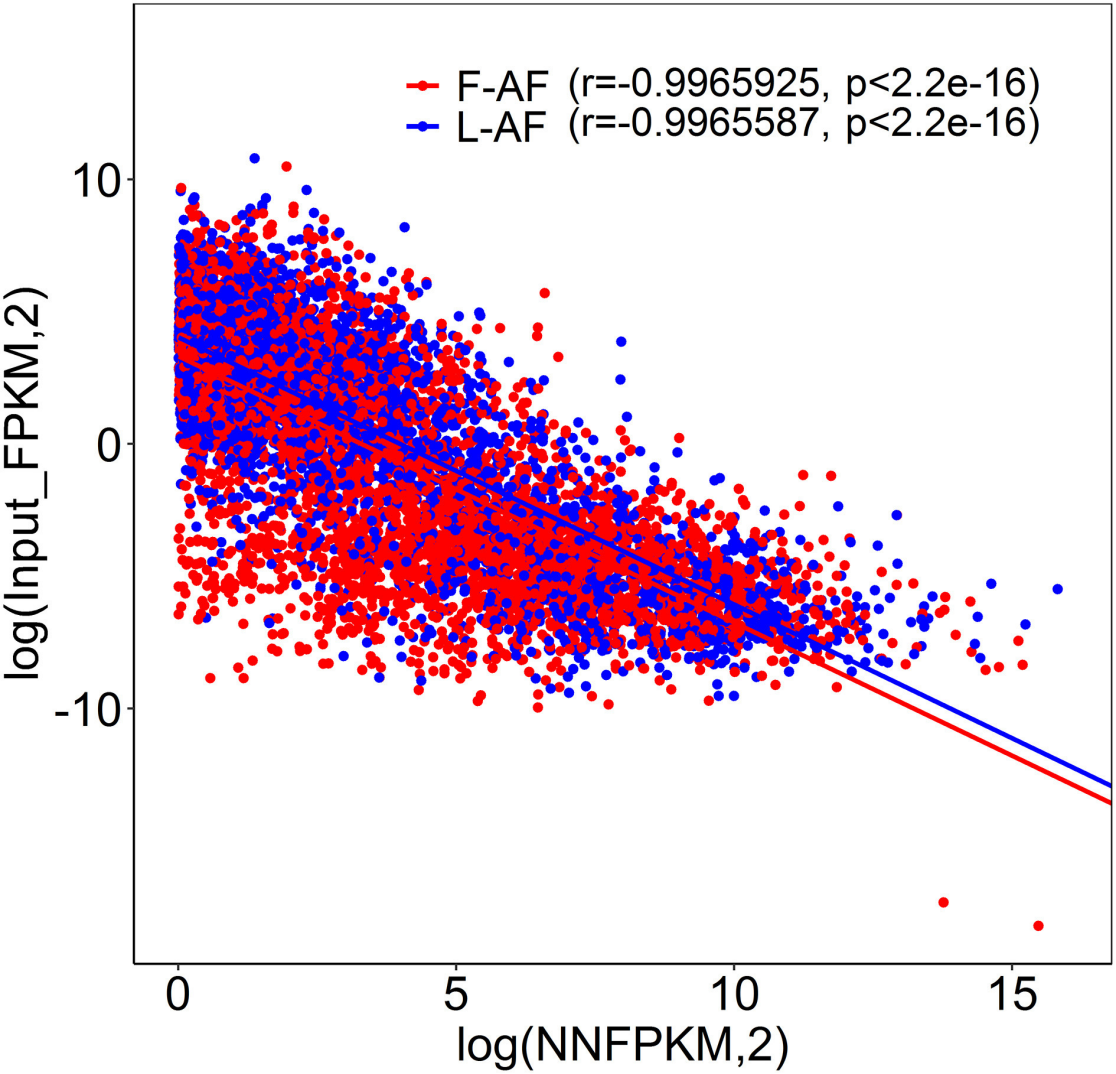
Plot of m^6^A peak enrichment and mRNA abundance in fat and lean chicken lines. Obvious negative correlation between m^6^A peak enrichment and modified mRNA abundance was found.

To understand whether m^6^A modification can affect gene expression, we used the input RNA-seq data to investigate the differential expression of genes between the two chicken lines. In total, 352 high expression genes and 424 low expression genes in the fat line compared with the lean line were identified (Supplementary Figure 2, Supplementary Data 8). Of the 1,172 line-dynamic m^6^A genes in total, 146 (12.5%) showed mRNA-expression differences (Supplementary Data 9), indicating that the mRNA levels of these genes may be regulated by m^6^A modification. Among the 146 genes, it should be noted that the mRNA levels of 95% (52/55) of the high m^6^A methylated genes were high and were named “hyper-up” genes. Similarly, the mRNA levels of 92% (84/91) of the low m^6^A methylated genes were low and were named “hypo-down” genes. Only 10 of the 146 genes (7%) showed opposing mRNA expression and m^6^A-methylation trends, and these genes were termed hyper-down or hypo-up genes (Figure 6A). Interestingly, several lipogenesis-related genes showed differences in both m^6^A methylation and mRNA expression. For instance, acyl-CoA synthetase long-chain family member 1 (*ACSL1*), which is associated with fatty acid transport, showed significantly higher m^6^A methylation and mRNA levels in the fat birds than in the lean birds (Figure 6B); while lipin1 (*LPIN1*), which is associated with adipocyte differentiation, exhibited lower m^6^A methylation and mRNA expression levels in the fat birds than in the lean birds (Figure 6C). Finally, we further examined whether gene regulation in the chicken adipose tissue is correlated with the m^6^A modification by plotting the abundance of m^6^A peaks with the mRNA expression levels. As shown in Figure 7, the plot of m^6^A peak enrichment level vs. mRNA abundance revealed a negative correlation between global RNA methylation and gene expression in both chicken lines (L-AF: Pearson's *r* = −0.9966, *P* < 0.0001; F-AF: Pearson's *r* =-0.9966, *P* < 0.0001).

## Discussion

Adipose tissue is important for energy storage, endocrine functions, and the control of energy metabolism (McGown et al., 2014; Choe et al., 2016). Over the past few decades, the regulatory mechanisms of adipose tissue development and fat deposition, such as transcription factors, DNA methylation and histone modification, have been extensively studied, and a series of important progressions have been made (Farmer, 2006; Wang et al., 2010; Zhu et al., 2012). Recently, various chemical modifications of RNA, such as m^6^A, *N*^1^-methyladenosine (m^1^A), and 5-methylcytosine (m^5^C), have been reported to play important roles in many physiological and pathological processes, including embryonic development, spermatogenesis, and the occurrence and development of a variety of diseases (Lin et al., 2017; Yang et al., 2019; Zhao et al., 2019). However, the role and underlying mechanism of RNA modification in adipose deposition are still uncharted territory. To this end, we conducted m^6^A methylome profiling of chicken adipose tissue using MeRIP-seq. To our knowledge, this is the first comprehensive high-throughput study to explore RNA modification in poultry adipose tissue. Our findings show the differences in m^6^A-modification patterns between the adipose tissue of fat and lean broilers. Further analysis suggested that m^6^A methylation may be an important factor in chicken adipose deposition via the regulation of gene expression.

Nearly 77 and 67% of mRNA transcripts underwent m^6^A methylation in L-AF and F-AF on average, respectively, suggesting that m^6^A plays a major role in adipose tissue development and fat deposition. In addition, the m^6^A peaks were primarily found in the highly conserved sequence motif GGACU (Figure 1B). M^6^A is generated by the binding of m^6^A methyltransferase to a highly conserved consensus sequence, GGACU (Shen et al., 2016). The RNA binding motif of METTL3, METTL14 and WTAP are GGAC, GGAC, and GACU, respectively (Liu et al., 2014). When the highly conserved GAC was mutated to GAU, m^6^A was no longer methylated in Rous sarcoma virus mRNA transcript (Kane and Beemon, 1987). A recent study showed that single nucleotide polymorphisms located at the GGAC positions could affect m^6^A methylation status (Zhang et al., 2020). Despite GGACU motif is important for the recognition by m^6^A methyltransferase, only a portion of GGACU sites are methylated *in vivo* (Gilbert et al., 2016), suggesting that the molecular mechanism regulating m^6^A modification needs to be further explored. In this study, interestingly, the m^6^A peaks were abundant not only in the CDS, stop codons, and 3′UTRs but also near the start codons (Figure 1D). This m^6^A-enrichment pattern is inconsistent with that of mammalian species (Meyer et al., 2012; Tao et al., 2017) but is similar to that of *Xenopus laevis* and *Arabidopsis thaliana* (Luo et al., 2014; Sai et al., 2020). This phenomenon may be attributed to differences in lipogenesis patterns between mammalian and birds (Gondret et al., 2001). It also seems to reflect the unique position of birds in the long evolutionary history of m^6^A modification in animals. In general, the predominance of m^6^A near stop codons and 3′UTRs has been found in most of the mRNAs of mammals, birds, amphibians, and plants (Luo et al., 2014; Tao et al., 2017; Fan et al., 2019; Sai et al., 2020), and this m^6^A-enrichment pattern may be representative of the typical mRNA m^6^A topology of eukaryotes. The high levels of m^6^A methylation in the 3′UTRs or near stop codons may be responsible for mRNA stability and alternative polyadenylation (Shen et al., 2016; Yue et al., 2018). Previous studies showed that m^6^A methylation in the CDS is likely to be associated with alternative splicing and translation efficiency (Zhao et al., 2014; Lin et al., 2019). Furthermore, the high m^6^A levels near the start codon may prevent mRNA degradation (Luo et al., 2014). In the present study, a negative relationship was observed between the global mRNA expression level and m^6^A methylation extent in chicken, which indicates m^6^A might affect chicken fat deposition at least in part through the regulation of mRNA stability.

The results of the GO and KEGG analyses in this study showed that m^6^A genes common to both broiler lines were significantly enriched in the processes and pathways associated with adipose development and fat deposition, such as lipid metabolism and the Wnt-signaling pathway (Figures 2B,C), which is consistent with a previous study that showed that the genes commonly methylated in the backfat of both fat (Jinhua) and lean (Landrace) pigs were mainly involved in cellular lipid metabolic processes (Wang et al., 2018). This result supports the findings of the previous study, in which mRNA m^6^A modifications were relevant to tissue-specific functions (Li et al., 2014). In addition, the L-AF-unique m^6^A genes were primarily enriched in developmental-associated processes and, intriguingly, in “ribosome” (Figures 3A,B), which includes genes such as ribosomal protein S10 (*RPS10*), ribosomal protein L10a (*RPL10A*), and mitochondrial ribosomal protein L16 (*MRPL16*). This result is different from the previous study, which showed that the unique m^6^A genes in the backfat of fat Jinhua pigs were significantly involved in translational initiation and ribosomal large-subunit biogenesis (Wang et al., 2018). This phenomenon may be attributed to differences in lipogenesis patterns between mammalian and avian species (Gondret et al., 2001). However, F-AF-unique m^6^A genes were significantly enriched in “mRNA processing” (Figure 4A), which includes genes such as pre-mRNA-processing factor 19 (PRPF19), cleavage- and polyadenylation-specific factor 6 (CPSF6), and CWC22 spliceosome-associated protein homolog (CWC22). The regulation of RNA metabolism by m^6^A modification depends on the recognition and binding of the specific m^6^A-reader proteins to the m^6^A sites (Yang et al., 2018). The m^6^A-reader protein YTH-domain-containing 1 (YTHDC1) has been shown to mediate alternative splicing, and YTH N6-methyladenosine RNA-binding protein 1 (YTHDF1) is responsible for enhancing translation efficiency (Yang et al., 2018). From our results, we speculated that the methylation of the mRNAs associated with mRNA processing and ribosome function might affect the expression of these same mRNAs, resulting in subsequent changes to global pre-mRNA splicing and protein synthesis, which might be another level of regulation involving alternative splicing and translation.

In mammals, an abundance of evidence has shown that m^6^A modification is involved in the regulation of adipose development and fat deposition (Zhang et al., 2015; Wu et al., 2017; Zong et al., 2019). Intriguingly, the results of the GO and KEGG analyses of the genes harboring dynamic methylated peaks showed that the high m^6^A methylated genes (fat line vs. lean line) were mainly involved in processes and pathways associated with lipid metabolism, such as fatty acid metabolism and fatty acid biosynthesis (Figure 5B), which further supports the importance of m^6^A in obesity. For example, stearoyl coenzyme A desaturase (SCD) is related to fatty acid metabolism and was up-methylated approximately 52-fold in the fat birds compared with the lean birds. SCD is a rate-limiting enzyme that catalyzes the formation of unsaturated fatty acids (Ntambi, 1999). A genome-wide association study in pigs identified *SCD* as a major gene affecting fatty acid composition and intramuscular fat content (Ros-Freixedes et al., 2016), and a recent study showed that *SCD* may be important for chicken adipose deposition and metabolism (Mihelic et al., 2020). It worth mentioning that insulin-like growth factor binding protein 2 (IGFBP2) was up-methylated about 20-fold in the fat birds compared with the lean birds (Table 4), although IGFBP2 is not involved with the pathways associated with lipid metabolism. IGFBP2 is a cytokine secreted by differentiating white adipocytes and is regulated by DNA methylation in human abdominal obesity (Zhang et al., 2019). In a previous study, we found that *IGFBP2* polymorphism (1196C>A) is significantly associated with AFW and AFP in the NEAUHLF population (Leng et al., 2009). In addition, we also found that the SNP 1196C>A within *IGFBP2* 3′UTR could influence its expression by affecting the regulation of gga-miR-456-3p (Yu et al., 2014). In this study, the m^6^A peak was found to be located in the CDS of *IGFBP2*, not 3**′**UTR. So we considered that the SNP 1196C>A may not affect the m^6^A methylation of *IGFBP2*. In contrast to the high m^6^A methylated genes, the genes with low m^6^A peaks (fat line vs. lean line) were primarily related to developmental-associated processes, such as cardiovascular system development (Figure 5C), further reinforcing the theory that there is a close relationship between abdominal obesity and cardiovascular disease (Sahakyan et al., 2015). Therefore, we postulated that the different m^6^A-methylation patterns might reflect significant phenotypic changes between the fat and lean broiler lines.

The mRNA m^6^A modifications are recognized and bound by m^6^A-reader proteins, which include YTH-domain family member 2 (YTHDF2) and insulin-like growth factor 2 mRNA-binding proteins (IGF2BPs) and are involved in the regulation of mRNA stability. YTHDF2 was shown to mediate mRNA decay (Zhu et al., 2014), and IGF2BPs (including IGF2BP1/2/3) are responsible for enhancing mRNA stability (Huang et al., 2018). In the present study, the mRNA expression levels of many genes were found to be affected by their m^6^A levels (Figures 6A, 7). Based on this result, we speculated that the m^6^A sites in the mRNA transcripts in chicken adipose tissue might be recognized and bound by YTHDF2 or IGF2BPs, thus changing the mRNA stabilities. However, further research is required to validate this hypothesis.

Our results show that several important lipogenic genes, including *ACSL1*, fatty acid synthase (*FASN*), *LPIN1*, and LDL receptor related protein 4 (*LRP4*), showed variations in both m^6^A methylation and mRNA expression. The expansion of adipose tissue mass is the result of an increase in the number of adipocytes and an increase in the size of individual fat cells. The number of adipocytes is determined by adipocyte differentiation (adipogenesis), while the size of the adipocytes is related to triglyceride (TG) accumulation in lipid droplets (Rosen and Spiegelman, 2006). ACSL1 is an acyl-CoA synthetaseand a long-chain fatty acid transport protein that can promote fatty acid uptake by adipocytes (Schaffer and Lodish, 1994; Tong et al., 2006). FASN is a key rate-limiting enzyme in *de novo* synthesis of fatty acids (Song et al., 2018). When there is excess energy in the body, most newly synthesized fatty acids are esterified into TGs for storage (Song et al., 2018). Our findings showed that the m^6^A methylation and mRNA expression of *ACSL1* and *FASN* were higher in the fat birds compared with the lean birds, indicating that hypermethylation of *ACSL1* and *FASN* mRNA in the fat line might promote the formation of TGs by enhancing mRNA stability and, thus, increasing gene-expression levels. Several lines of evidence have shown that adipocytes are integral to energy metabolism regulation (Rondinone, 2006). Preadipocyte differentiation is controlled by a complex network of multiple transcriptional regulators, of which peroxisome proliferator-activated receptor gamma (PPARγ) is the most important (Farmer, 2006). Research has shown that LPIN1 interacts with and enhances the transcriptional activity of PPARγ and promotes the differentiation of 3T3-L1 preadipocytes (Kim et al., 2016). In the current study, the m^6^A methylation and mRNA expression of *LPIN1* were higher in the lean birds compared with the fat birds, which is consistent with a previous study that found *LPIN1* expression levels were increased in the adipose tissue of lean subjects compared with the fat subjects (van Harmelen et al., 2007) This phenomenon might be due to the compensatory increase in *LPIN1* expression to maintain the balance of energy metabolism in the lean birds. We also found 10 genes showing opposing mRNA expression and m^6^A methylation patterns (Figure 6A). Interestingly, one of these, *LRP4*, is related to lipid metabolism; *LRP4* mRNA was low methylated and its expression level was high in the fat line compared with the lean line. LRP4 is a transmembrane protein of the low-density lipoprotein receptor family (Alrayes et al., 2020). A recent study showed that the mice knockout of *LRP4* gene in adipocytes exhibit a reduction in adipocyte size and improved lipid and glucose homeostasis (Kim et al., 2019), suggesting that LRP4 is a positive regulator of adipocyte size. Therefore, it is conceivable that the hypomethylation of *LRP4* in the fat line might increase adipocyte size by promoting *LRP4* mRNA levels through a reduction in YTHDF2-mediated mRNA decay, although, further exploration is needed to shed light on this aspect. We propose that the m^6^A modifications within the mRNAs of *ACSL1, FASN, LPIN1*,and *LRP4* may be closely involved in adipose deposition and energy homeostasis in chickens. It is notable that, of the 1,172 line-dynamic m^6^A genes, most (87.5%) did not show mRNA-level variations between the fat and lean broiler lines. This phenomenon may be due to two reasons: (1) m^6^A may affect chicken abdominal fat deposition via other mechanisms, such as translation regulation, in addition to the regulation of mRNA stability; (2) Gene expression regulation is complex. Besides m^6^A mehthylation, the mRNA level of gene is influenced by various transcription and posttranscriptional regulatory factors, such as transcription factors (Farmer, 2006), transcription cofactors (Fabre et al., 2012), DNA methylation (Zhu et al., 2012), histone modification (Wang et al., 2010), chromatin remodeling (Siersbaek et al., 2011), and non-coding RNAs (Li et al., 2016; Losko et al., 2018).

## Conclusion

In summary, we analyzed the m^6^A methylomes of chicken abdominal adipose tissues and proposed that m^6^A modification may play a key role in regulating the expression of genes contributing to lipid metabolism and adipogenesis. This comprehensive m^6^A map not only provides a basis for studying the roles of m^6^A methylation in chicken fat deposition but also opens a new avenue in the study of RNA epigenetics in adipobiology.

## Data Availability Statement

The datasets presented in this study can be found in online repositories. The names of the repository/repositories and accession number(s) can be found below: [NCBI SRA AND PRJNA657377].

## Ethics Statement

The animal study was reviewed and approved by The Ministry of Science and Technology of the People's Republic of China (Approval Number: 2006-398) and were approved by the Laboratory Animal Management Committee and the Institutional Biosafety Committee of Northeast Agricultural University (Harbin, China).

## Author Contributions

BC contributed to the design of the experiments, carried out the experiments, performed the statistical analyses, and prepared the manuscript. LL contributed to the design of the experiments and helped with managing the birds. ZL, WW, YL, YJ, NW, and HL contributed to writing the manuscript. SW conceived and designed the study, participated in data interpretation, and contributed to writing the manuscript. BC and LL contribute equally to this study. All authors gave final approval for publication.

## Conflict of Interest

The authors declare that the research was conducted in the absence of any commercial or financial relationships that could be construed as a potential conflict of interest.
