# Supplementary figures and images for: Profiling of RNA N6-Methyladenosine Methylation Reveals the Critical Role of m6A in Chicken Adipose Deposition
Source: Front Cell Dev Biol. 2021 Feb 5;9:590468. doi: 10.3389/fcell.2021.590468 (PMC7892974; doi:10.3389/fcell.2021.590468)

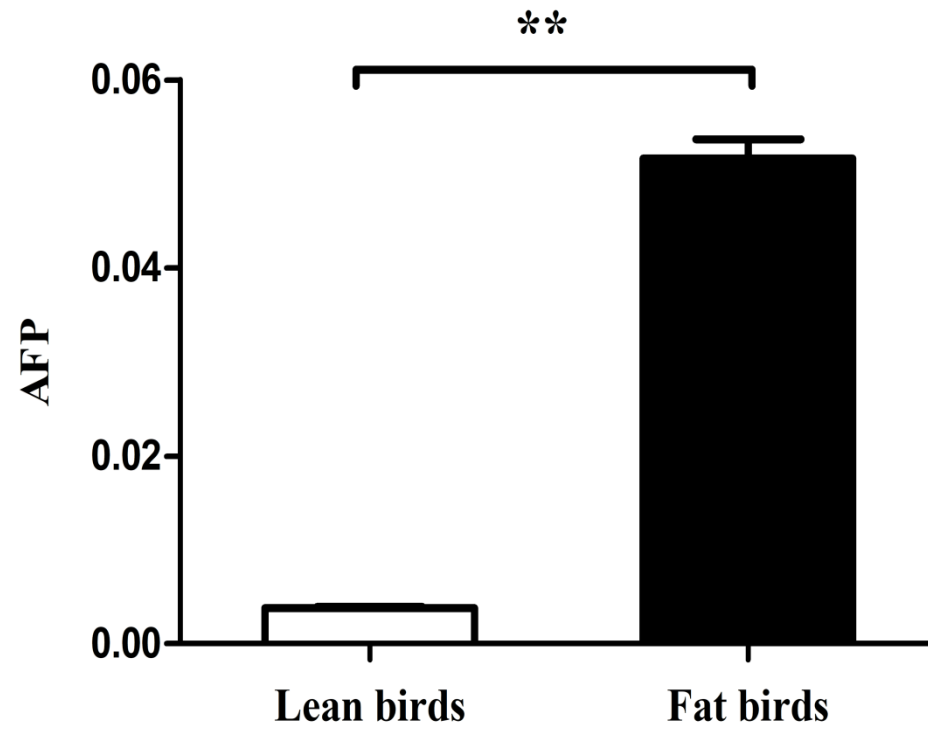

Supplement: Supplementary file 1 [file Data_Sheet_1.PDF]

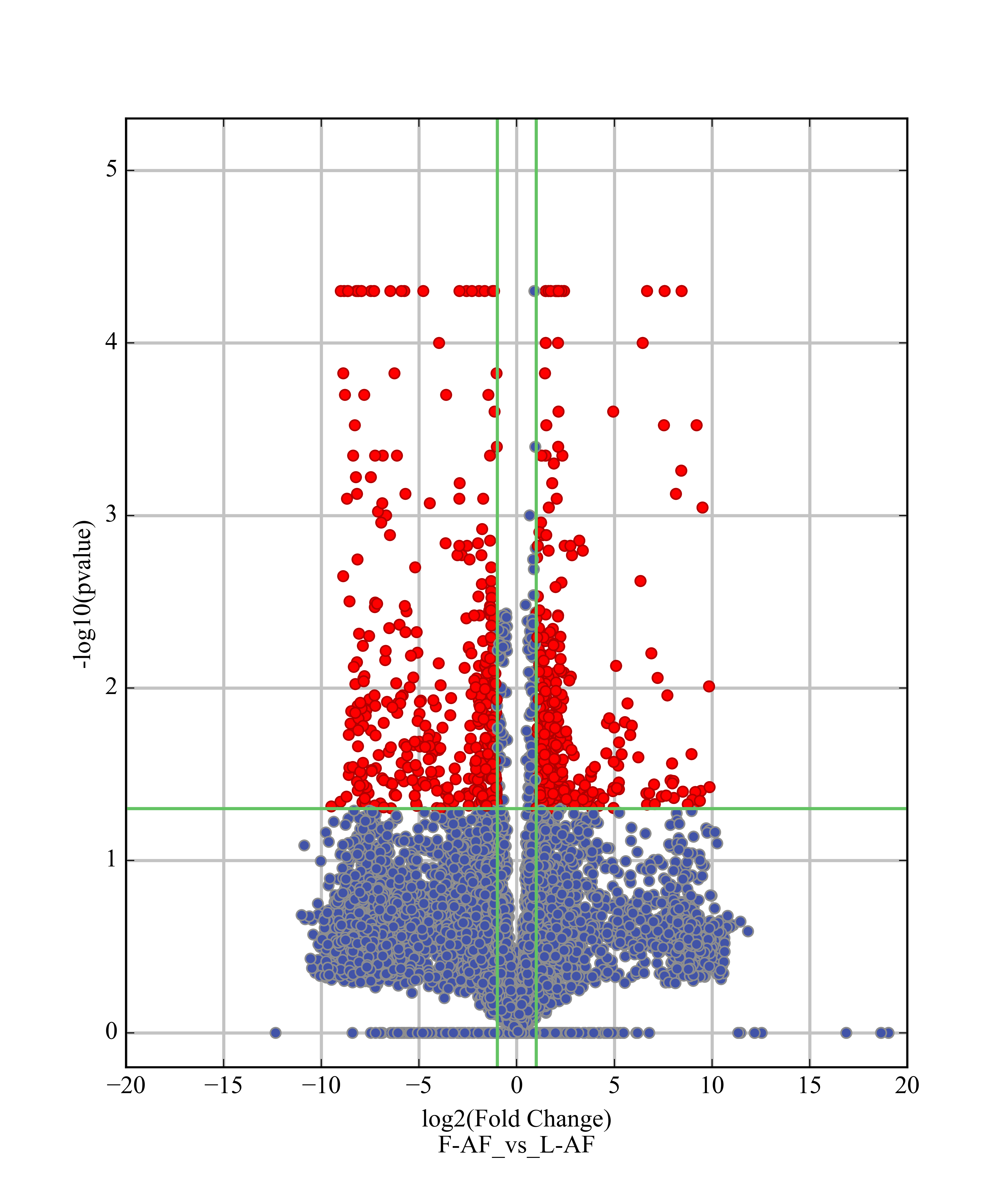

Supplement: Supplementary file 2 [file Image_1.TIFF]
