# Supplementary material for: Profiling of RNA N6-Methyladenosine Methylation Reveals the Critical Role of m6A in Chicken Adipose Deposition
Source: Front Cell Dev Biol. 2021 Feb 5;9:590468. doi: 10.3389/fcell.2021.590468 (PMC7892974; doi:10.3389/fcell.2021.590468)
Supplement: Supplementary file 12 [file Table_1.docx]

**Supplementary Material**

**Profiling of RNA *N^6^*-methyladenosine methylation reveals the critical role of m^6^A in chicken adipose deposition**

**Bohan Cheng^1,2,3,^**^†^**, Li Leng^1,2,3,^**^†^**, Ziwei Li^1,2,3^, Weijia Wang^1,2,3^, Yang Jing^1,2,3^, Yudong Li^1,2,3^,Ning Wang^1,2,3^, Hui Li^1,2,3^ and Shouzhi Wang^1,2,3,*^**
*^1^ Key Laboratory of Chicken Genetics and Breeding, Ministry of Agriculture and Rural Affairs, Harbin 150030, Heilongjiang, China*

*^2^ Key Laboratory of Animal Genetics, Breeding and Reproduction, Education Department of Heilongjiang Province, Harbin 150030, Heilongjiang, China*

*^3^College of Animal Science and Technology, Northeast Agricultural University, Harbin 150030, Heilongjiang, China*

^*^Corresponding author's e-mail address: shouzhiwang@neau.edu.cn

^†^Bohan Cheng and Li Leng contribute equally to this study.

**Fig. S1.** Comparison of AFP of the birds used in the study.***P*<0.01. AFP, abdominal fat percentage.

**Fig. S2.** Volcano map of differentially expressed genesbetween the two chicken lines.

**Data S1.**m^6^A peaks in abdominal adipose tissues from fat and lean chicken lines. (XLSX 9393 KB)

**Data S2.**Common and line-unique m^6^A genes. (XLSX 85 KB)

**Data S3.** GO biological process enrichment of common and line-unique m^6^A genes. (XLSX 233 KB)

**Data S4.** KEGG enrichment of common and line-unique m^6^A genes. (XLSX 87 KB)

**Data S5.** Line-dynamicm^6^A genes. (XLSX 405 KB)

**Data S6.** GO biological process enrichment of line-dynamicm^6^A genes. (XLSX 47 KB)

**Data S7.** KEGG enrichment of line-dynamicm^6^A genes. (XLSX 45KB)

**Data S8.** Differentially expressed genes between the two chicken lines. (XLSX 250KB)

**Data S9.** Genes that showing the difference in both m^6^A methylation and mRNA expression between the two chicken lines. (XLSX 5210 KB)
